# Exploiting PRMT5 as a target for combination therapy in mantle cell lymphoma characterized by frequent *ATM* and *TP53* mutations

**DOI:** 10.1038/s41408-023-00799-6

**Published:** 2023-02-17

**Authors:** Yuxuan Che, Yang Liu, Yixin Yao, Holly A. Hill, Yijing Li, Qingsong Cai, Fangfang Yan, Preetesh Jain, Wei Wang, Lixin Rui, Michael Wang

**Affiliations:** 1 Department of Lymphoma and Myeloma, The University of Texas MD Anderson Cancer Center, 1515 Holcombe Blvd., Houston, TX 77030 USA; 2 Department of Bioinformatics and Computer Biology, The University of Texas MD Anderson Cancer Center, 1515 Holcombe Blvd., Houston, TX 77030 USA; 3 School of Biomedical Informatics, University of Texas Health Science Center at Houston, 7000 Fannin Street, Houston, TX 77030 USA; 4 Department of Medicine, the University of Wisconsin-Madison, 1111 Highland Avenue, Madison, WI 53726 USA; 5 Department of Stem Cell Transplantation and Cellular Therapy, The University of Texas MD Anderson Cancer Center, Houston, TX 77030 USA

**Keywords:** B-cell lymphoma, Cancer therapeutic resistance

## Abstract

Constant challenges for the treatment of mantle cell lymphoma (MCL) remain to be recurrent relapses and therapy resistance, especially in patients harboring somatic mutations in the tumor suppressors *ATM* and *TP53*, which are accumulated as therapy resistance emerges and the disease progresses, consistent with our OncoPrint results that *ATM* and *TP53* alterations were most frequent in relapsed/refractory (R/R) MCL. We demonstrated that protein arginine methyltransferase-5 (PRMT5) was upregulated in R/R MCL, which predicted a poor prognosis. PRMT5 inhibitors displayed profound antitumor effects in the mouse models of MCL with mutated *ATM* and/or *TP53*, or refractory to CD19-targeted CAR T-cell therapy. Genetic knockout of PRMT5 robustly inhibited tumor growth in vivo. Co-targeting PRMT5, and ATR or CDK4 by using their inhibitors showed synergistic antitumor effects both in vitro and in vivo. Our results have provided a rational combination therapeutic strategy targeting multiple PRMT5-coordinated tumor-promoting processes for the treatment of R/R MCL with high mutation burdens.

## Introduction

MCL is an incurable and aggressive subtype of non-Hodgkin B-cell lymphoma [1, 2]. Major advances have led to the identification of clinically effective targeted therapies against MCL, including inhibitors of Bruton’s tyrosine kinase (BTK) and Bcl-2 [3, 4]. CD19-directed CAR T-cell therapy (brexucabtagene autoleucel) demonstrated remarkable efficacy in BTK inhibition-resistant MCL patients [5]. Unfortunately, most MCL patients experience recurrent relapses [6, 7]; therefore, novel therapeutic agents and combination therapies need to be continuously developed.

MCL frequently accompanies with a high degree of genomic instability and multiple somatic mutations in DNA damage repair (DDR) pathways, especially in ataxia-telangiectasia-mutated (*ATM)* and *TP53* genes [8–10]. The t(11;14) (q13;q32) translocation that juxtaposes *CCND1* to the *IGH* gene, a hallmark of MCL, leads to the overexpression of cyclin D1 [11, 12], that promotes cell cycle progression and global transcriptional regulation [13]. *CDKN2A* and *MTAP* on chromosome 9p21.3 are frequently co-deleted in MCL, especially in ibrutinib (IBN)-resistant tumors [14]. The *CDKN2A* gene encodes for the tumor suppressor p16^INK4A^ protein, a negative regulator of CDK4/6. Loss of p16^INK4A^ leads to sustained activation of CDK4/6, resulting in uncontrolled proliferation [15].

PRMT5 is a type-II arginine methyltransferase and forms a tight hetero-octameric complex with its substrate-binding partner MEP50 (encoded by *WDR77*) [16]. Along with pICln, PRMT5 and MEP50 form the 20S methylosome in the cytoplasm which symmetrically dimethylates spliceosome proteins prior to their assembly [17]. PRMT5 symmetrically dimethylates histones [18], transcription factors [19, 20], and chromatin remodeling factors in the nucleus [21]. Importantly, PRMT5 activates homologous recombination via regulating alternative splicing of TIP60 [22], indicating that DNA damage-prone cells with compromised DDR pathways should be more sensitive to PRMT5 inactivation/depletion. In addition, PRMT5 directly or indirectly modulates p53, c-Myc, cyclin D1, and CDK4/6 [19, 23, 24] while the cyclin/kinases reciprocally augment PRMT5 oncogenic functions [25]. PRMT5 upregulation also leads to the suppression of p53 through arginine methylation [26], making targeting PRMT5 an insightful approach to reactivate p53 tumor suppressor function. As a cell survival and proliferation protein, PRMT5 overexpression or overactivation has been implicated in several human cancers, including MCL [27–29].

*ATM* is the most frequently mutated gene in the MCL mutational landscape [10]. Coordinated with the tumor suppressor *RB1* and *TP53*, the ATM kinase plays a central role in the cellular response to DNA double-strand breaks. *ATM* gene defects in MCL patients frequently resulted in a complete inactivation of ATM [30]. *TP53* mutations have been frequently observed in MCL with blastoid/pleomorphic histology [31]. Loss of *ATM* abrogates p53 activation in response to DNA damage, allowing the cells with unrepaired DNA to escape from p53 surveillance [32]. To explore the therapy strategies for R/R MCL, especially those with multiple defects on key genes such as *ATM* and *TP53*, we screened numerous promising compounds targeting DDR, p53 signaling, and cell cycle, including PRMT5 inhibitors, for their cytotoxicity on MCL cell lines with different genetic backgrounds.

In this study, we presented evidence to demonstrate frequent genetic alterations in *ATM* and *TP53* genes in most R/R patients with MCL, including those with intrinsic or acquired resistance to ibrutinib or CD19-targeted CAR T-cell therapy. MCL cells with frequent mutations in genes including *ATM* or *TP53* are sensitive to PRMT5 inhibition or depletion. Using our preclinical cell line-derived xenograft (CDX) and patient-derived xenograft (PDX) models, our study has identified a rational combination therapeutic strategy which acts to restrain the dysregulated DNA damage response and cell cycle progression in MCL.

## Materials and methods

### Cell lines and primary patient cells

The MCL cell lines JeKo-1, Mino, Z-138, Maver-1, Rec-1, JVM-2, JVM-13, UPN1, HBL-2, SP-49, and the human lymphoblastoid cell line (LCL) were obtained from the American Type Culture Collection. The Granta-519 cell line was obtained from the Leibniz-Institute DSMZ. Peripheral blood, bone marrow, biopsies, and apheresis specimens were collected from patients with MCL after consent and approval from the Institutional Review Board at the University of Texas MD Anderson Cancer Center.

### Cell cytotoxicity and proliferation assay

Cell cytotoxicity screening was conducted as described previously [33], using CellTiter-Glo Cell viability Assay Reagent (Promega). For proliferation assay, cells were seeded at 2 × 10^5^ cells per well in 24-well plates and treated with indicated inhibitors for 5 d. The live cells were counted each day.

### Cell apoptosis assay

Cells were seeded at 2 × 10^5^ cells per well in 24-well plates and incubated with indicated inhibitors for various periods of time, then stained with annexin-V (BD Biosciences) and propidium iodide (Invitrogen). Apoptosis was determined using flow cytometry on NovoCyte Flow Cytometer (ACEA Biosciences), and the data were analyzed with FlowJo v10.

### Cell cycle analysis

Cell cycle analysis was done as previously described [34]. The cell cycle phases were quantified and analyzed on a Novocyte Flow Cytometer (ACEA Biosciences).

### Reactive oxygen species (ROS) measurement

ROS level in MCL cells was measured as described previously [33], using H2DCFDA-cellular ROS detection probe (Invitrogen) and flow cytometry.

### Western blotting assay

Western blotting was performed as described previously [33]. The antibodies were obtained from Cell Signaling Technology (PRMT5, p21 Waf1/Cip1, GAPDH, ATM, phospho-histone H2AX (Ser139), cyclin D1, Rb, phospho-Rb (Ser780), CDK6, PARP, cleaved caspase-7), Invitrogen (CDK4), Sigma (H4R3me2s, SYM10, SYM11), Santa Cruz (p53, β-actin), and Bethyl (HdmX/MDM4).

### MDM4 RNA splicing analyses by RT-PCR

Total RNA was extracted from treated MCL cell lines using Qiagen RNeasy Mini Kit. First-strand cDNA was synthesized using the iScript Reverse Transcription Supermix. cDNA was amplified with MDM4 exon-specific primers as described elsewhere [35].

### Real-time qPCR

Total RNA was extracted from treated MCL cell lines and cDNA was prepared as described above. Expression of specific mRNAs was determined by qPCR using the SYBR green supermix (Bio-Rad) as described elsewhere [35].

### Whole-exome sequencing

Selected specimens from patients with MCL were subjected to whole-exome sequencing (WES) and the raw output from Illumina was analyzed for somatic mutations, functional annotation, and expression of mutant alleles, as described previously [33].

### Immunofluorescence staining

Immunofluorescence staining was performed as previously described [33]. Images were acquired on Zeiss microscope Axio Observer 7 and ZEN image analysis software. The number of γH2AX foci was then quantified using ImageJ.

### Immunohistochemistry

Tumor microarrays (TMA) were obtained from the MD Anderson Tissue Bank after consent and approval from the Institutional Review Board at the University of Texas MD Anderson Cancer Center and used for immunohistochemistry as described elsewhere [36]. The following antibodies were used for immunohistochemistry staining using the Dako Autostainer with appropriate positive and negative controls: PRMT5 (Cell Signaling Technology), H4R3me2s (Sigma), and Ki67 (Invitrogen). Immunohistochemistry (IHC) images were semi-quantitated for 3,3’-diaminobenzidine (DAB) intensity using ImageJ (Fiji). The correlation between the indicated protein levels was determined by the Pearson chi-square test. *P* values were determined by chi-square test (F), or Student’s *t* test using the Prism software.

### Targeted metabolomics analysis

Metabolites extracted from either ibrutinib-sensitive or -resistant MCL cell lines were subjected to targeted metabolomics analysis for nucleic acids and amino acids and analyzed as described previously [37].

### CRISPR/Cas9-mediated genome editing

Gene knockout in MCL cell lines was achieved using CRISPR/Cas9 genome editing, as described previously [38].

### Patient-derived xenograft (PDX) or cell line-derived xenograft (CDX) mouse models

All experimental protocols were approved by the Institutional Animal Care and Use Committee of The University of Texas MD Anderson Cancer Center. NSG mice at 6–8 weeks old were injected subcutaneously with MCL cell lines for CDX mouse models, or patient-derived MCL cells for PDX. The mice were treated when the tumors became palpable. Tumor volumes were measured on specified days and calculated using the formula: volume = length × width^2^ × 0.5, and tumor growth was monitored until humane endpoints.

### Statistical analysis

All assays were performed in triplicate and expressed as mean ± SEM or SD. The statistical significance of differences was determined by Student’s *t* test, two-way ANOVA, or nonlinear regression analysis. Overall survivals were calculated with the Kaplan–Meier method and compared with the log-rank test.

## Results

### MCL is characterized by the most frequent somatic mutations in *ATM* and *TP53* genes

**Fig. 1:**
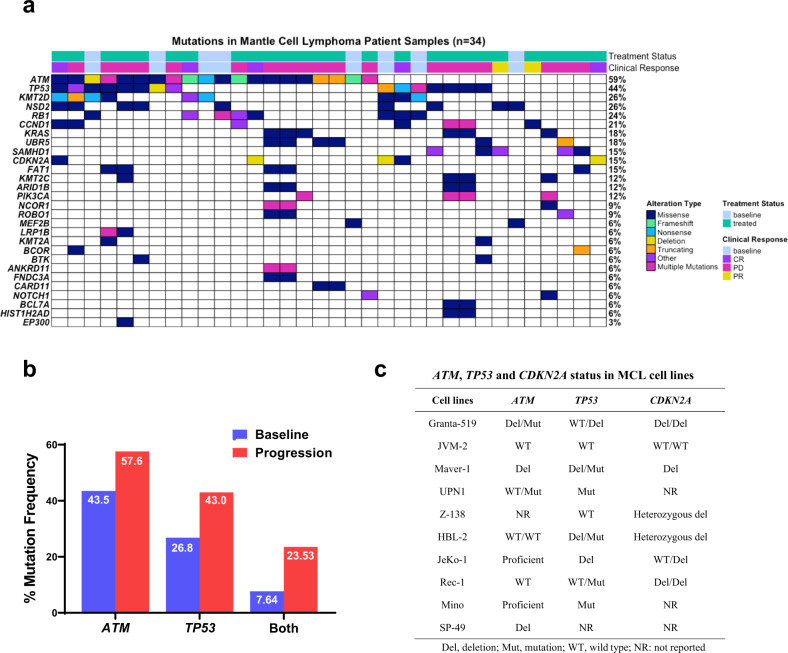
MCL is characterized by the most frequent somatic mutations in *ATM* and *TP53* genes. **a** OncoPrint showing the mutational spectrum in 34 patients with MCL from two studies, of whom 19 patients progressed or transformed on ibrutinib treatment. Each column represents a patient tumor sample. The clinical response and treatment status are annotated on the top track. The OncoPrint displays the somatic mutations and copy number alterations identified by targeted gene sequencing. Genes with nonsynonymous mutations or copy number alterations in two or more patients are listed. The numbers on the right side represent the percentages of mantle cell lymphoma (MCL) tumors carry mutations or copy number alterations of each specific gene. CR complete response, PR partial response, PD progressive disease. **b** Meta-analysis comparing the mutational frequencies of *ATM* and *TP53* between MCL patients at baseline and those at disease progression. **c** Genetic status of *ATM*, *TP53*, and *CDKN2A* in MCL cell lines as reported in the literature.

With an initial objective to decipher the molecular mechanisms of therapy resistance and disease progression in MCL, we reanalyzed our previously published whole-exome sequencing (WES) datasets for the mutational frequency of genes in 34 MCL patient specimens from two studies [9, 33]. Among them, 19 patients progressed or transformed after ibrutinib treatment. *ATM* and *TP53* alterations were the most frequent in this 34 MCL patients (59% and 44%, respectively) (Fig. 1a). Among them, 50% (*ATM*) and 60% (*TP53*) were missense mutations, with the remaining annotated as frameshift, deletion, truncation, nonsense, and multiple mutations, which are predicted to lead to loss or gain of functions to the genes. Noteworthy, among the top 10 most frequently mutated, *KMT2D*, *NSD2*, *RB1*, *CCND1*, and *CDKN2A* are known to play important roles in the lymphomagenesis and disease progression of MCL. We next performed a meta-analysis on the public datasets from multiple MCL studies containing a total of 2008 evaluable MCL samples at either baseline or disease progression [39] and found the frequencies of concurrent mutations in *ATM* and *TP53* at disease progression are apparently higher than that at baseline (Fig. 1b). Incorporating the data from both OncoPrint and Meta-analysis, the result suggests that functional disruptions in these tumor suppressors may be associated with therapy resistance and disease progression in MCL. *ATM* and *TP53* mutations are also reported frequently present in MCL cell lines (Fig. 1c).

### Cytotoxicity screening reveals DDR and cell cycle inhibitors are effective in inducing cell death in *ATM*- or *TP53*-mutated MCL

**Fig. 2:**
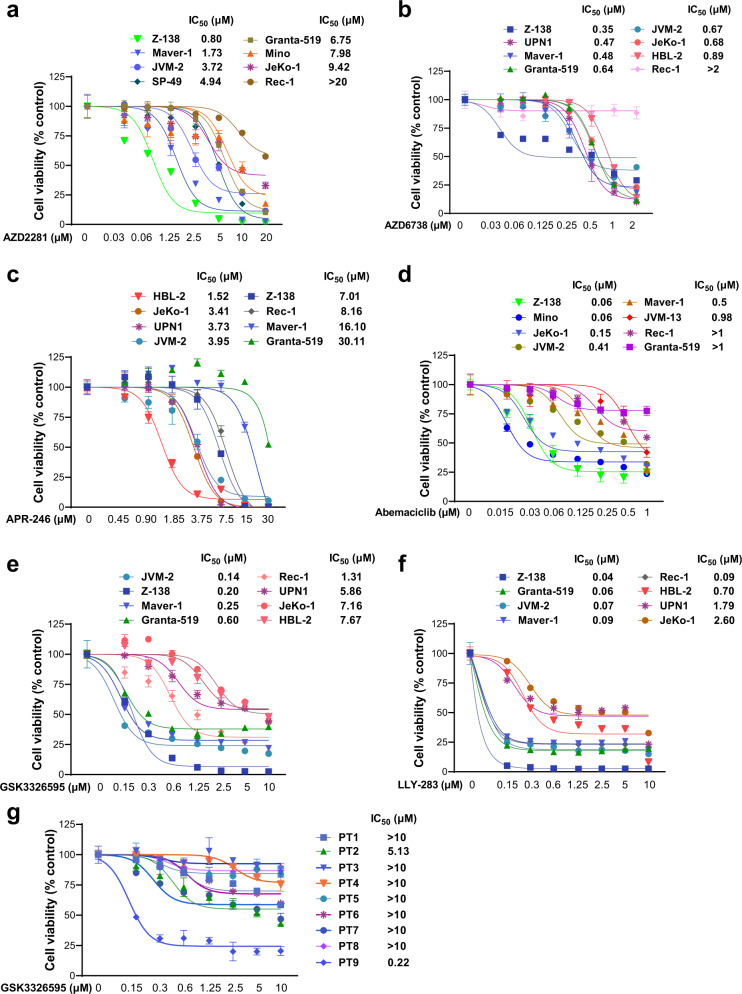
Cytotoxicity screening reveals DDR and cell cycle inhibitors are effective in inducing cell death in *ATM*- or *TP53*-mutated MCL. MCL cell lines were treated with a two-fold serial dilution of **a** PARP inhibitor AZD2281, **b** ATR inhibitor AZD6738, **c** p53 reactivator APR-246, or **d** CDK4/6 inhibitor abemaciclib for 3 days and the viability were evaluated using CellTiter-Glo. Similarly, the viability of MCL cells treated with a two-fold serial dilution of **e** PRMT5 inhibitors GSK3326595 or **f** LLY-283 for 6 days were evaluated due to longer responding time required for PRMT5 inhibitors. **g** Viability of primary MCL cells from R/R patients treated with a two-fold serial dilution of PRMT5 inhibitors GSK3326595 for only 3 days. The primary cells stop growing after a few days in culture, therefore we shortened the treatment period.

Activations of the DDR and cell cycle signaling pathways are the most significant hallmarks in MCL. To identify a potential strategy for the treatment of R/R MCL, we performed cytotoxicity screening of inhibitors targeting DDR and cell cycle promotors in MCL cell lines with various mutations in *ATM*, *TP53,* and *CDKN2A* (Fig. 1c). PARP and ATR inhibitors were tested here because cancer cells with *ATM* deficiency are sensitive to PARP and ATR inhibition [40–42]. Treatment of MCL cell lines with the PARP inhibitor AZD2281 as a single agent exerted cytotoxicity with IC_50_ values ranging from 0.8 μM to >20 μM (Fig. 2a), with no clear cytotoxicity preference to a certain subgroup of those cell lines. Impressively, the ATR-specific inhibitor AZD6738 displayed potent cytotoxicity with IC_50_ values in a nanomolar range in most of the tested MCL cells, regardless of the status of *ATM* (Fig. 2b). APR-246 is a first-in-class small molecule to facilitate normal conformation and function of mutated *TP53*. Dose-response cytotoxicity analysis with APR-246 as a single agent displayed IC_50_ values above 3 μM for most MCL cell lines tested, with a poor correlation to the *TP53* status (Fig. 2c). Upregulated CDK4 signaling in the *CDKN2A*-deficient MCL cells represents another potential therapeutic target [43]. Treatment with CDK4/6 inhibitor abemaciclib induced cell death with IC_50_ values ranging from 60 nM to over 1 μM (Fig. 2d). However, abemaciclib failed to show increased cytotoxicity to *CDKN2A*-deficient MCL cells. Considering that protein arginine methylation plays an important role in regulating DNA repair genes [22], we evaluated the effect of PRMT5 inhibition on MCL growth. PRMT5 inhibitor GSK3326595, currently in phase I clinical trial for solid tumors and non-Hodgkin’s lymphoma (NCT02783300; ClinicalTrials.gov), acts by occupying the binding site of substrate peptides in the PRMT5/MEP50 complex and thereby inhibits the enzymatic activity of PRMT5. *ATM*-deficient (Granta-519 and Maver-1) and/or *CDKN2A*-deficient (Granta-519, Maver-1, and Rec-1) MCL cell lines are sensitive to PRMT5 inhibitor GSK3326595 (Fig. 2e). Similarly, treatment with another PRMT5 inhibitor LLY-283 also exerted potent cytotoxicity with lower IC_50_s (0.04-0.09 μM) in *ATM*-deficient and/or *CDKN2A*-deficient cell lines (Fig. 2f). Interestingly, JVM-2 cells with wild-type *ATM*, *TP53*, and *CDKN2A*, were also sensitive to both PRMT5 inhibitors. The reduction in the enzymatic activity was confirmed by a dramatic reduction in the level of symmetric dimethylarginine (SDMA)-modified proteins and H4R3me2s (histone H4R3 dimethyl symmetric), an arginine methylated substrate of the enzyme, in cells treated with the inhibitors (Supplementary Fig. S1a). PRMT5 inhibition substantially reduced c-Myc methylation (Z-138) detected by SDMA-specific antibodies, or the overall protein level (Granta-519, JVM-2, and Mino) (Supplementary Fig. S1b), in agreement with the recent finding that PRMT5 is required for lymphomagenesis triggered by multiple oncogenic drivers [44]. Of note, PRMT5 inhibition with GSK3326595 or EPZ015666 (another PRMT5 inhibitor acting in a non-SAM-competitive but peptide substrate-competitive manner) also resulted in remarkably reduced cell proliferation in MCL cell lines (Supplementary Fig. S1c, d). However, unlike the established MCL cell lines, treatment with GSK3326595 on primary lymphoma cells from MCL patients at progression resulted in modest growth inhibition (Fig. 2g), possibly due to the short time of treatment, short survival or unknown mechanism of resistance. However, the effect of PRMT5 inhibition could be better assessed in the PDX models where tumors are established from primary MCL cells.

### PRMT5 is upregulated in R/R patients with MCL, and its high expression is positively associated with poor prognosis

**Fig. 3:**
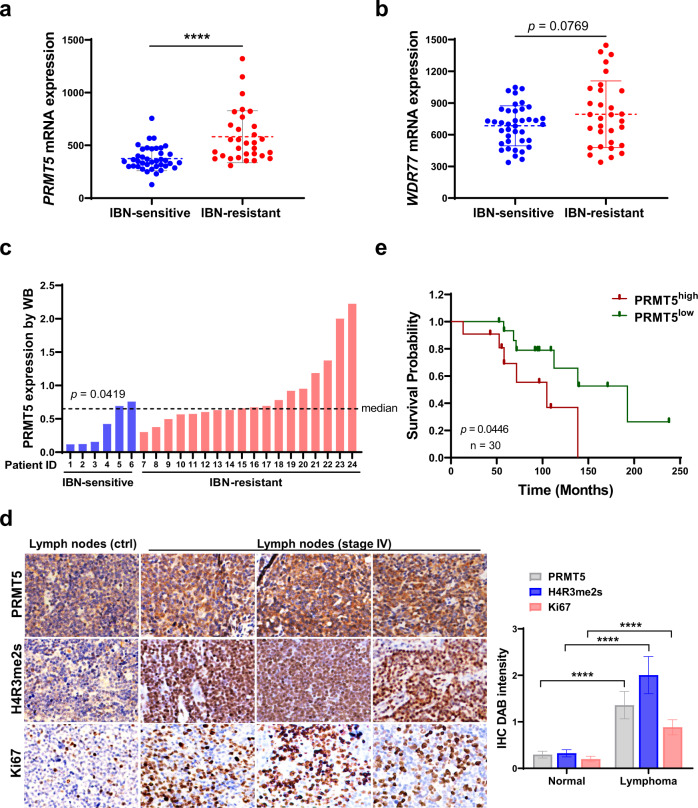
PRMT5 is upregulated in R/R patients with MCL, and its high expression is positively associated with poor prognosis. **a**, **b** PRMT5 (**a**) and WDR77 (**b**) expression analyzed by RNA-seq in clinical specimens from unpaired ibrutinib-sensitive or -resistant patients with MCL. **c** PRMT5 protein expression was determined by western blotting in clinical specimens from MCL patients sensitive or resistant to ibrutinib treatment. The PRMT5 bands were quantitated using ImageJ and then normalized with a corresponding loading control. **d** Left: Immunohistochemistry analysis of MCL tissue microarray (TMA) for the expression of PRMT5, H4R3me2s, and proliferation marker Ki67. Representative images of tissue staining are shown. Right: Quantification of PRMT5, H4R3me2s, and Ki67 staining signal by ImageJ was shown. *P* value was determined by two-tailed independent Student’s *t* test. Correlation between the indicated protein levels was determined by Pearson chi-square test. *r* correlation coefficient. **e** Kaplan survival analysis of MCL patients with high or low PRMT5 expression. For all panels: *P < 0.05; **P < 0.01; ***P < 0.001; ****P < 0.0001; ns (not statistically significant), *P* ≥ 0.05.

To probe the relationship between the expression of PRMT5 and disease progression in MCL, we performed RNA-seq analysis on clinical specimens collected from MCL patients treated with ibrutinib. PRMT5 and its substrate-binding partner MEP50 (encoded by *WDR77*) were substantially upregulated in ibrutinib-resistant MCL cells (Fig. 3a, b) along with that of PRMT1 and PRMT7 (Supplementary Fig. S2a, b). Immunoblotting analyses of clinical specimens including apheresis, bone marrow, and lymph node confirmed the overexpression of PRMT5 protein in MCL (Fig. 3c and Supplementary Fig. S2c). In addition, PRMT5 expression was highly upregulated in a panel of MCL cell lines compared to the human lymphoblastoid cell line (Supplementary Fig. S2d). Consistently, immunohistochemical analysis exhibited strong PRMT5 staining in late-stage MCL tissues compared to the normal lymph node. IHC images from a total of 26 individual patient samples were used to evaluate the average relative expression of PRMT5, H4R3me2s and Ki67 expression. High expression levels of PRMT5 were positively correlated with its substrate H4R3me2s and cell proliferation marker Ki67, with a correlation coefficient (R) value of 0.9129 and 0.9797, respectively (Fig. 3d), depicting the cell proliferation and tumor-promoting function of PRMT5. Targeted metabolomics analysis displayed elevated S-adenosyl-methionine (SAM) and metabolite S-adenosyl-homocysteine (SAH), a cofactor and metabolite of PRMT5, respectively, in ibrutinib-resistant MCL cell lines (Supplementary Fig. S2e). Importantly, Kaplan–Meier survival analysis demonstrated that the high expression of PRMT5 at the transcriptional level was associated with poorer overall survival of patients with MCL (Fig. 3e). These data suggest that PRMT5 could be exploited as a potential therapeutic target, especially for R/R MCL with high mutation burdens.

### PRMT5 inhibition or genetic ablation induces accumulation of DNA damage in *ATM*-deficient MCL cell lines and restores the expression and tumor-suppressive function of wild-type p53

**Fig. 4:**
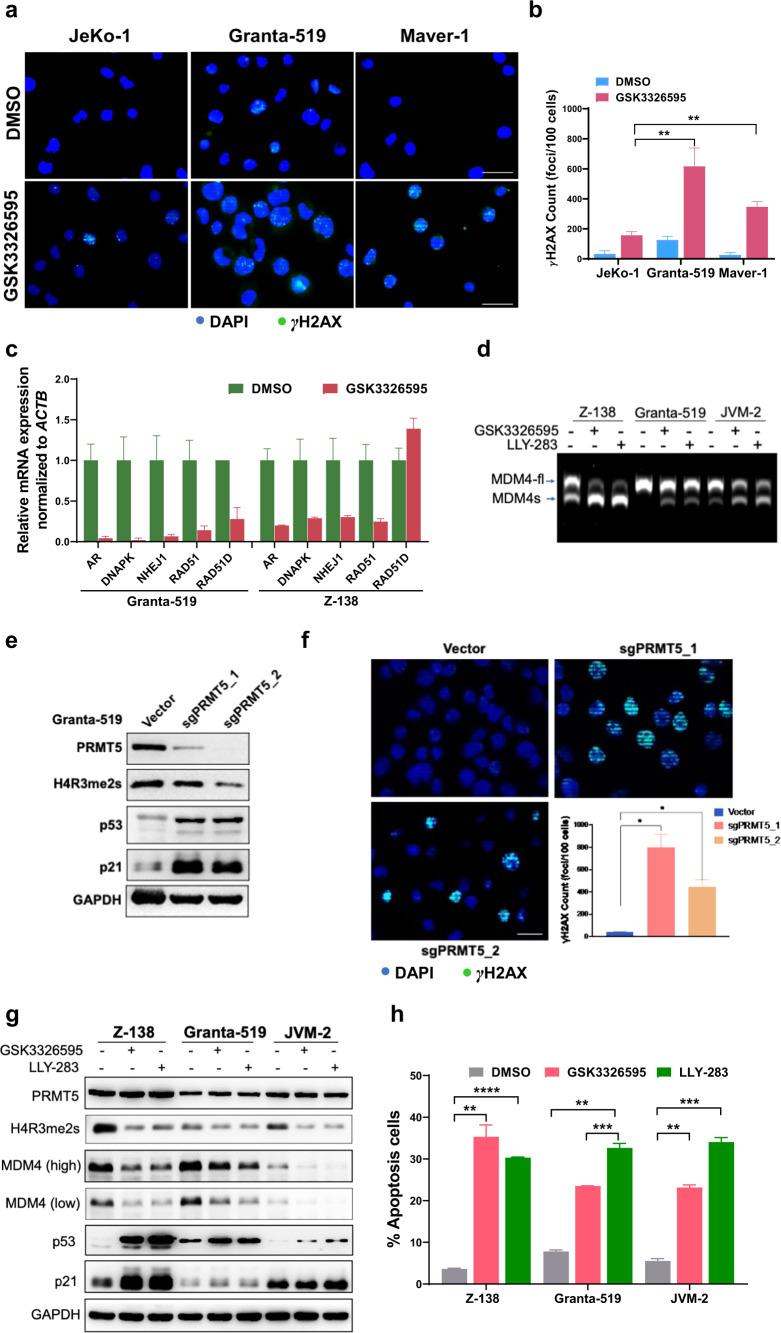

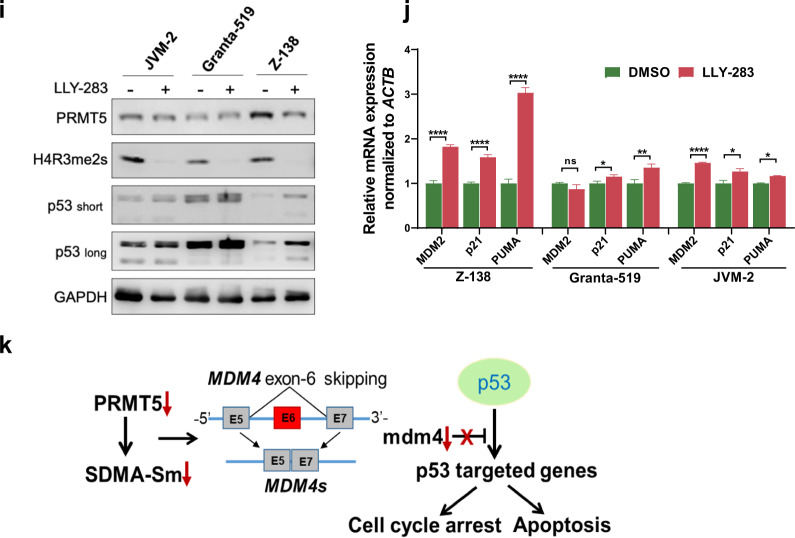
PRMT5 inhibition or depletion induces accumulation of DNA damage in *ATM*-deficient MCL cell lines and restores the expression and tumor-suppressive function of wild-type p53. **a** Indicated MCL cell lines were treated with PRMT5 inhibitor GSK3326595 and subjected to IF staining with anti-γH2AX, a marker for DNA damage. Scale bars = 20 μm. **b** γH2AX foci in (**a**) were quantified using ImageJ. **c** Expression of genes (AR, DNAPK, NHEJ1 and RAD51) in DDR pathways analyzed by real-time qPCR in Granta-519 and Z-138 cell lines treated with PRMT5 inhibitor GSK3326595. **d** Indicated cell lines were treated with PRMT5 inhibitors GSK3326595 (1 µM) and LLY-283 (1 µM) and subjected to RT-PCR analysis of MDM4 mRNA splicing. **e** Expression of PRMT5 and p53 pathway genes determined by immunoblotting in Granta-519 with or without PRMT5 KO. **f** Granta-519 cells were stained with anti-γH2AX for 2 h after exposure to 10 Gy γ-irradiation. Scale bars = 20 μm. γH2AX foci were quantitated using ImageJ. **g** Indicated cell lines were treated as in (**d**) before they were subjected to western blotting analysis of MDM4 and p53. **h** Indicated MCL cell lines were treated with PRMT5 inhibitor GSK3326595 or LLY-283 and subjected to apoptosis analysis. **i** Western blotting analysis of the expression of PRMT5 and p53 in the cell lines treated as in (**h**). **j** Expression of p53 target genes (MDM2, p21, and PUMA) analyzed by real-time qPCR in in Z-138, Granta-519 and JVM-2 cell lines treated with LLY-283. **k** A schematic illustration of the alternative splicing of MDM4 upon PRMT5 depletion stabilizing p53. For all panels: *P < 0.05; **P < 0.01; ***P < 0.001; ****P < 0.0001; ns (not statistically significant), *P* ≥ 0.05.

Activation of the DDR pathways and cell cycle progression are among the most significant hallmarks in MCL, and the genetic alterations in these pathways are enriched in ibrutinib-resistant MCL [10]. *ATM* and *TP53* mutations or deletion found in MCL patient specimens have also been reported in several MCL cell lines, particularly deletion of *ATM* with gain-of-function mutations of *TP53* (Maver-1) and deletion or mutations on both *ATM* alleles with heterozygous deletion of *TP53* (Granta-519) (Fig. 1c and Supplementary Table S1). Loss of *ATM* was shown to abolish p53 activation in response to DNA damage [32]. As PRMT5 has been implicated in epigenetic, post-transcriptional, and post-translational regulation of DDR genes [45, 46], we hypothesized that *ATM*-deficient MCL cells were sensitive to DNA damage inducers when PRMT5 function was blocked. Indeed, PRMT5 inhibition with GSK3326595 resulted in more accumulated unrepaired DNA damage, as evidenced by an increase in the number of γH2AX foci (Fig. 4a). The average count of γH2AX foci per 100 cells was significantly higher in *ATM*-deficient MCL cell lines (617 in Granta-519 and 347 in Maver-1) than in *ATM*-proficient JeKo-1 (157) (Fig. 4b). This result was supported by downregulation of DDR genes (AR, DNAPK, NHEJ1, and RAD51) upon PRMT5 inhibition in Granta-519 and Z-138 cell lines (Fig. 4c), along with elevated oxidative stress marker, the reactive oxygen species (ROS) (Supplementary Fig. S3a). To validate our observations, we generated PRMT5-knockout Granta-519 clones using CRISPR/Cas9 and examined the contribution of PRMT5 to DNA damage-repairing capacity in the cells. As a result, the depletion of PRMT5 dramatically increased the accumulation of DNA fragmentation and p53/p21 expression, compared to the control (Fig. 4e, f).

Activation of p53 is essential for functional ATM/p53 signaling. In addition to phosphorylation by ATM at multiple sites that stabilizes p53, p53 accumulation is complexly modulated by other post-translational modifications, including ubiquitination by MDM2 and MDM4, two negative regulators of p53, leading to its degradation. The MDM4 alternative splicing events including exon skipping are considered as sensors of PRMT5 depletion and defects in the constitutive splicing machinery [47]. To interrogate the role of PRMT5 upregulation on p53 expression in MCL, we treated MCL cell lines expressing wild-type p53 (Z-138, Granta-519, and JVM-2) with GSK3326595 and LLY-283. The result indicated that PRMT5 ablation induced the alternative splicing of MDM4 mRNA by exon 6 skipping, leading to the production of a functionally defective short form of splicing variant as shown by RT-PCR analysis (Fig. 4d). As expected, decreased expression or functionally loss of MDM4 upon PRMT5 inhibition stabilized p53 and restored its function, as illustrated by an increased level of p53 and its downstream p21, a negative regulator of cyclin-dependent kinases (CDKs), in all 3 cell lines tested (Fig. 4g). As a functional outcome for p53 restoration, treatment with PRMT5 inhibitors significantly induced apoptosis in the above 3 cell lines (Fig. 4h and Supplementary Fig. S3b) along with increased expression of p53 (Fig. 4i) and mRNA expression of p53 target genes (MDM2, p21, and PUMA) (Fig. 4j), suggesting that PRMT5 promotes the tumor survival and growth for MCL through downregulation of p53, as illustrated in the schematic diagram (Fig. 4k).

Since the p53 reactivator APR-246 has been reported to selectively induce apoptosis in other cancer cells in a p53 status-dependent manner [48], we assessed its effect in MCL cell lines. Surprisingly but consistent with our cytotoxicity results above, APR-246 dosed at 30 µM failed to induce apoptosis in several ibrutinib-resistant MCL cell lines (Maver-1 and Granta-519), with only modest effect in Z-138 and JVM-2. The effect seemed independent of *TP53* status in MCL, as the drug also induced massive apoptosis in *TP53*-deficient JeKo-1 cell (Supplementary Fig. S3c). The results insinuate that more potent and specific therapeutics are needed to stabilize wild-type p53 or reactivate mutant p53.

### PRMT5 inhibition or genetic ablation attenuates MCL tumor growth in both CDX and PDX models

**Fig. 5:**
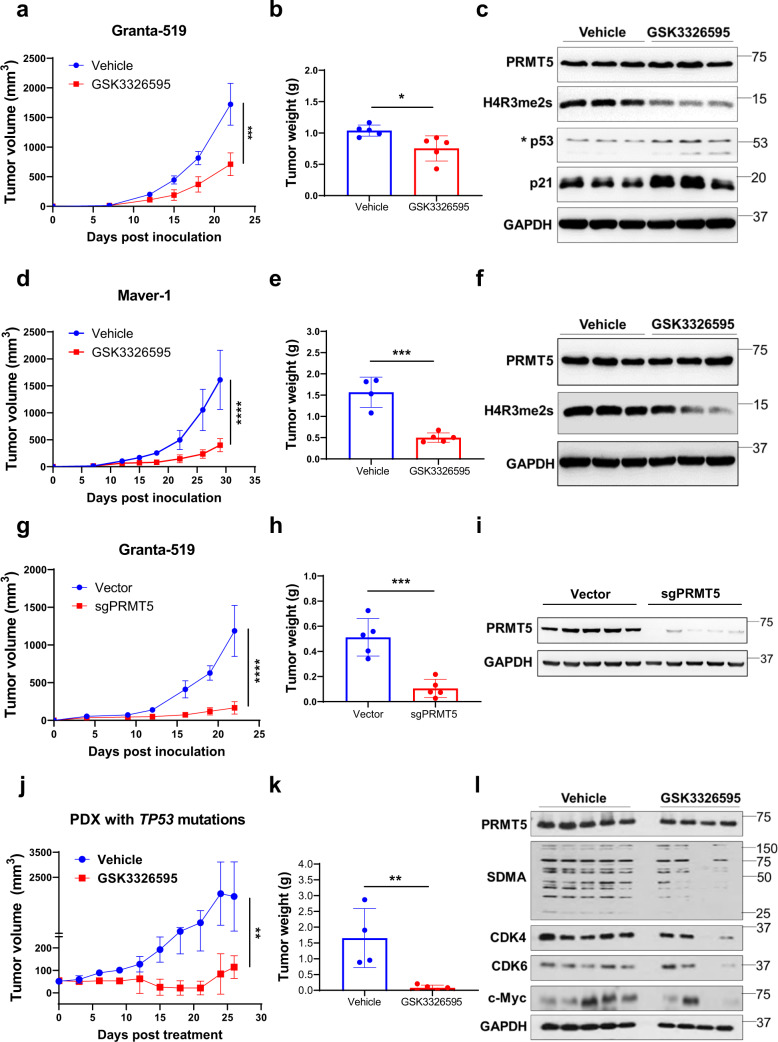

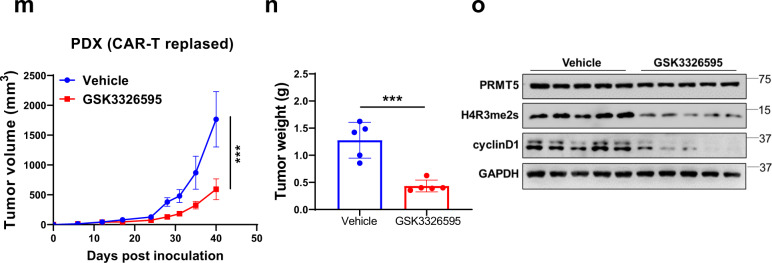
PRMT5 inhibition or depletion attenuates MCL tumor growth in both CDX and PDX models. NSG mice xenografts implanted with **a** Granta-519 or **d** Maver-1 were treated with either vehicle solvent or PRMT5 inhibitor GSK3326595 (100 mg/kg, daily). Tumor volumes were measured and plotted against the time of treatment (*n* = 5). Statistical significance between vehicle and PRMT5 inhibitor treatment was determined by a two-way ANOVA test. Tumor weights of **b** Granta-519 and **e** Maver-1 xenografts from (**a**) and (**d**), respectively, were measured at the endpoint of treatment. Statistical significance between vehicle and PRMT5 inhibitor treatment was determined by a two-tailed independent Student’s *t* test. **c**, **f** Western blotting analysis of the expression of PRMT5, H4R3me2s, p53, and p21 in xenograft tumors from (**a**) and (**d**), respectively. **g** Granta-519 cells with or without PRMT5 KO were injected into NSG mice. Tumor volume was monitored and plotted. Statistical significance between vehicle and PRMT5 inhibitor treatment was determined by two-way ANOVA test. **h** Tumor weights at the endpoint are presented. Statistical significance between vehicle and PRMT5 inhibitor treatment was determined by two-tailed independent Student’s *t* test. **i** PRMT5 expression in tumors of mice was confirmed using western blotting. **j** PDX models were derived from a MCL patient who had *TP53* mutations. The PDX mice were treated with vehicle or GSK3326595 (100 mg/kg, daily). Tumor sizes were measured at different days and plotted relative to the number of days post treatment (*n* = 5). Statistical significance between vehicle and PRMT5 inhibitor treatment was determined by a two-way ANOVA test. **k**, **l** Tumor weight measurement, statistical analysis, and western blotting were performed as described in (**b**) and (**c**). **m** PDX models derived from a MCL patient who had relapsed after CD19 CAR T-cell therapy were treated with GSK3326595 (100 mg/kg, daily). Tumor sizes were measured at different days and plotted against the time of treatment (*n* = 5). Statistical significance between vehicle and PRMT5 inhibitor treatment was determined by two-way ANOVA test. **n**, **o** Tumor weight measurement, statistical analysis, and western blotting were performed as described in (**b**) and (**c**). For all panels: *P < 0.05; **P < 0.01; ***P < 0.001; ****P < 0.0001; ns (not statistically significant), *P* ≥ 0.05.

To validate the target specificity of the PRMT5 inhibitors, we knocked out PRMT5 in three MCL cell lines (Maver-1, Granta-519, and Z-138) with different *TP53* and *ATM* status (Fig. 1c and Supplementary Table S1) using CRISPR-Cas9 gene editing and evaluated the cytotoxic effect of the inhibitors. The result confirmed that genetic ablation of PRMT5 rendered the cells significantly less or not sensitive to PRMT5 pharmacological inhibition with IC_50_ > 10 μM, compared to that of sgRNA-vector transduced cells, with IC_50_ ranging from 0.79–5.66 μM (GSK3326595) and 0.49–2.5 μM (LLY-283) (Supplementary Fig. S4a–d), indicating the effects observed with these inhibitors are target specific. The genetic ablation of PRMT5 resulted in remarkably reduced cell proliferation of Granta-519 (Supplementary Fig. S4e, f), consistent with the tumor proliferation suppressive effect of PRMT5 inhibitors (Supplementary Fig. S1c, d). To evaluate the in vivo antitumor efficacy of PRMT5 inhibitors, we generated two CDX mouse models using Granta-519 and Maver-1. Treatment of the tumor-bearing mice with GSK3326595 remarkably attenuated tumor growth (Fig. 5a, b, d, e and Supplementary Fig. S5d). The activity of the inhibitor was confirmed by the reduction in the level of H4R3me2s (Fig. 5c, f). To validate the antitumor effect of PRMT5 inhibitors in vivo, we also injected PRMT5-knockout Granta-519 into mice and the result confirmed that PRMT5 knockout robustly inhibited the tumor growth (Fig. 5g–i). To exploit the therapeutic potential for aggressive MCL, we established an in-house PDX model from an MCL patient who had *TP53* mutations and *MYC* rearrangement. Treatment with GSK3326595 significantly diminished tumor growth in the mice (Fig. 5j, k), accompanied by decreased H4R3me2s and downregulation of CDK4/6 in the drug-treated tumors (Fig. 5l). Importantly, using a PDX model generated from a patient with MCL who had *ATM* mutations and deletion of exon 7 in the *TP53* at diagnosis and relapsed after CD19-directed CAR T-cell therapy, we observed that PRMT5 inhibitor treatment markedly attenuated the tumor growth (Fig. 5m, n), with decreased methylation of PRMT5 substrate histone H4R3me2s (Fig. 5o). Of note, the expression of cyclin D1 was also decreased upon treatment, consistent with the report that PRMT5 may directly or indirectly positively modulate cyclin D1 [44]. Consistently, single-cell RNA-seq analysis displayed increased expression of *PRMT5* and *WDR77* (encoding MEP50) in clinical specimens from MCL patients who were resistant to ibrutinib or the CAR T-cell therapy (Supplementary Fig. S2f, g). This result implies that targeting PRMT5 may overcome resistance to ibrutinib or the CAR T-cell therapy in MCL with *ATM* or *TP53* alterations.

### Concurrent targeting of PRMT5 and DDR effectors exerts augmented antitumor efficacy towards MCL with *ATM* and/or *TP53* alterations

**Fig. 6:**
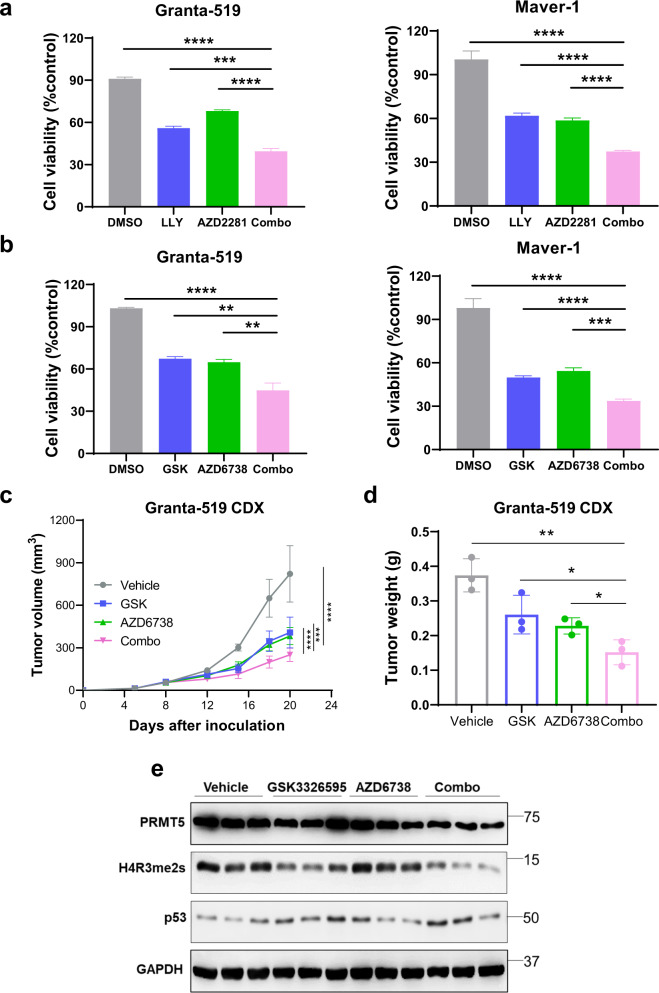
Concurrent targeting of PRMT5 and DDR effectors exerts augmented antitumor efficacy towards MCL with *ATM* and/or *TP53* alterations. **a** Cell viability of Granta-519 and Maver-1 cells treated with LLY-283 (LLY, 0.25 µM), AZD2281 (2.5 µM), or in combination. **b** Cell viability of Granta-519 and Maver-1 cells treated with GSK3326595 (GSK, 0.6 µM), AZD6738 (0.6 µM), or their combination. **c** NSG mice implanted with Granta-519 and treated with vehicle, GSK3326595 (GSK, 100 mg/kg, daily), AZD6738 (25 mg/kg, daily), or the combination of both inhibitors. Tumor volumes were measured on specified days. Average tumor volumes were plotted against the number of days after treatment (*n* = 5). Statistical significance values relative to inhibitors combination were determined by two-way ANOVA test. **d** Tumor weight measured at the endpoint of treatment. Statistical significance values relative to inhibitors combination was determined by two-tailed independent Student’s *t* test. **e** Western blotting analysis of the expression of PRMT5, H4R3me2s, and p53 in representative tumors from mice in (**d**). For all panels: *P < 0.05; **P < 0.01; ***P < 0.001; ****P < 0.0001; ns (not statistically significant), *P* ≥ 0.05.

To achieve more potent antitumor efficacy on MCL with *ATM* and/or *TP53* alterations, we performed dose-response cytotoxicity analysis of PRMT5 inhibitors in combination with DDR protein inhibitors. Combination treatment with PRMT5 and PARP inhibitors displayed a synergistic cytotoxic effect in *ATM*-deficient Granta-519 and Maver-1 cells (Fig. 6a). Targeting ATR may exploit synthetic lethality in cancer cells with impaired compensatory DDR through *ATM* loss, whether as monotherapy or combined with DNA-damaging drugs [41]. Impressively, combination treatment with PRMT5 and ATR inhibitors also showed more potent cytotoxicity on *ATM*-deficient MCL cells than those treated with either inhibitor alone, as indicated by the synergistic cytotoxic effects (Fig. 6b and Supplementary Fig. S5a). To evaluate the anti-lymphoma efficacy of the combination treatment in vivo, we choose a CDX model derived from *ATM*-deficient Granta-519 and treated the mice with inhibitors of PRMT5, and ATR, alone or together. Both inhibitors remarkably diminished tumor growth, and the combination treatment achieved enhanced tumor rejection than either of the single inhibitors (Fig. 6c, d), without any significant effect on mouse body weights (Supplementary Fig. S5b). Immunoblotting confirmed that GSK3326595 in combination with AZD6738 resulted in decreased levels of H4R3me2s but increased the expression of p53 in the drug-treated tumors compared to every single agent (Fig. 6e).

### Simultaneous targeting of PRMT5 and CDK4 suppresses cell cycle progression and tumor growth in *CDKN2A*-deficient MCL

**Fig. 7:**
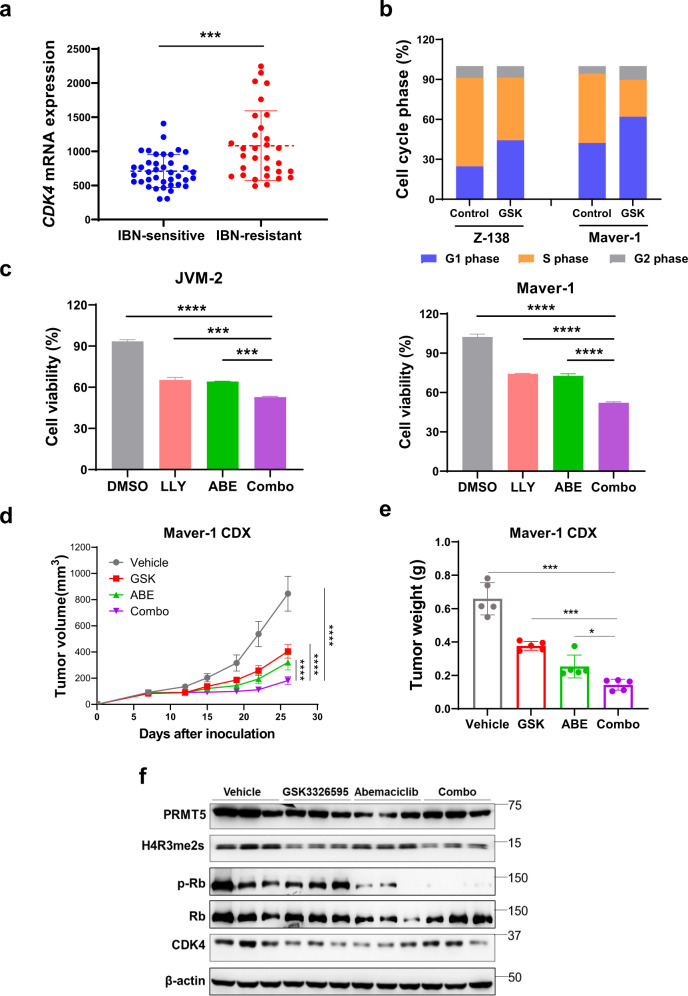
Simultaneous targeting of PRMT5 and CDK4 suppresses cell cycle progression and tumor growth in *CDKN2A*-deficient MCL. **a** Relative CDK4 expression levels analyzed using the RNA-seq dataset in Fig. 3a, b. **b** Indicated MCL cells treated with 1 μM GSK3326595 (GSK) and subjected to cell cycle analysis. **c** Cell viability of JVM-2 and Maver-1 cells treated with LLY-283 (LLY), abemaciclib (ABE), or the combination of both inhibitors. **d** Tumor volumes of Maver-1-derived xenografts treated with vehicle, GSK3326595 (GSK, 100 mg/kg, daily), abemaciclib (ABE, 10 mg/kg, daily), or the combination of both inhibitors were measured on specified days. Average tumor volumes were plotted against the number of days after treatment (*n* = 5). Statistical significance values relative to inhibitors combination was determined by two-way ANOVA test. **e** Tumor mass weight measured at the endpoint of treatment. Statistical significance values relative to inhibitors combination were determined by two-tailed independent Student’s *t* test. **f** Western blotting analysis of the expression of PRMT5, H4R3me2s, CDK4, and p-Rb in the representative tumors from (**e**). For all panels: *P < 0.05; **P < 0.01; ***P < 0.001; ****P < 0.0001; ns (not statistically significant), *P* ≥ 0.05.

RNA-seq analysis of specimens from MCL patients also revealed that CDK4 mRNA was significantly upregulated in ibrutinib-resistant MCL cells (Fig. 7a), in line with the observation of frequent deletion of *CDKN2A* in MCL (Fig. 1a). To examine the role of PRMT5 in the regulation of cyclin proteins and kinases, we first performed cell cycle analysis. Treatment of MCL cell lines with PRMT5 inhibitor-induced cell cycle arrest at the G1 phase in *CDKN2A*-deficient Z-138 and Maver-1 cells (Fig. 7b and Supplementary Fig. S5c). This result led us to further investigate if CDK4 inhibitors, which also induce G1-phase arrest, have a synergistic growth inhibition effect on MCL cells. The combination treatment exhibited synergistic cytotoxic effects in both *CDKN2A*-deficient (Maver-1) and -proficient (JVM-2) MCL cell lines (Fig. 7c). To assess the antitumor efficacy of the combination treatment in vivo, we choose the Maver-1-derived CDX model harboring both *TP53* mutations and deletion of *CDKN2A* and treated the mice with either GSK3326595, abemaciclib, or in combination. Each inhibitor remarkably attenuated tumor growth, whereas the combination treatment exhibited a more profound tumor suppression (Fig. 7d, e), with no significant effect on mouse body weights (Supplementary Fig. S5e). Western blotting confirmed decreased levels of H4R3me2s in GSK3326595-treated cells and decreased levels of p-Rb, a negative regulator of the G1/S transition in cell cycle, in abemaciclib-treated cells (Fig. 7f). These data suggest that co-targeting PRMT5 and CDK4/6 presents a new therapeutic option for MCL patients with a deletion of *CDKN2A*.

## Discussion

Among those derailed signaling, somatic mutations in DNA damage repair pathways, especially *ATM* and *TP53*, persist in MCL and continue to accumulate as the disease evolves [9]. Another hallmark of MCL is co-deletion of *CDKN2A* and *MTAP* on chromosome 9p21.3 [33]. Without p16, the protein product of *CDKN2A*, CDK4 loses its negative regulator and therefore over-activated. As a potent driver of cell malignancy, PRMT5 is involved in multiple cell survival and proliferation pathways including DDR, p53 activation, cell cycle regulation, and c-Myc in tumor cells through protein arginine methylation and downstream effects such as RNA splicing regulation. Examination of our clinical specimens confirmed that PRMT5 was significantly upregulated in MCL, especially in ibrutinib-resistant patients. High expression of PRMT5 predicted poor prognosis, presenting as another potential therapeutic target for MCL.

Although our data could not explicitly conclude that the sensitivities of MCL cells to PRMT5 inhibition are exclusively dependent on the particular genetic status, the PRMT5 inhibitors exerted more potent cytotoxicity in *ATM, TP53*, and/or *CDKN2A*-deficient CDX or PDX models, indicating that they are more dependent on PRMT5 which could be exploited as a potential therapeutic target for R/R MCL with heavy mutation burdens.

In this work, we primarily aimed at identifying potential therapeutic strategies for R/R MCL featured with frequent *ATM* and *TP53* mutations. Using CDX models, we present evidence that the combination of PRMT5 inhibitors with either DDR or cell cycle inhibitors significantly increased the anti-lymphoma potency in MCL with *ATM* and/or *TP53* lesions, attributing to the intrinsic interaction between PRMT5 and DDR pathways. PRMT5 promotes homologous recombination repairing during DNA damage via guiding alternative splicing of the key DNA repair protein TIP60 [22]. Our data and a previous report [47] also unraveled that PRMT5 guided alternative splicing of MDM4 (Fig. 4d), a negative regulator of p53, which induces its degradation by ubiquitination, and thus allows the cells with DNA damage to skip p53 surveillance system. In line with these findings, inhibition of PRMT5 resulted in the accumulation of DNA breaks (Fig. 4a) and stabilization of p53 (Fig. 4g). MCL cells with *ATM* deficiency could be more sensitive to PRMT5 inhibition than those with proficient *ATM*. In line with this hypothesis, we found that *ATM*-deficient PDX tumors (Fig. 5m, n) as well as cell lines Granta-519 and Maver-1 (Fig. 5a, b, d, e), are sensitive to PRMT5 inhibition. However, further investigation on isogenic cell lines with or without *ATM* deletion is required to clarify the relationship between PRMT5 and ATM. PRMT5 inhibitors have been reported less efficient in p53 knockout cells [49]. We also found that p53-null MCL cell line JeKo-1 is relatively less sensitive to PRMT5 inhibition (Fig. 2e, f). PRMT5 Inhibition in MCL cells led to G1-phase cell cycle arrest (Fig. 7b), which was likely resulted from DDR-p53-p21 axis activation (Fig. 4g). Anti-proliferation effect of PRMT5 inhibitors could be further augmented by CDK4/6 inhibition which induced G1-phase cell cycle arrest with a different mechanism.

In conclusion, our results provide a rational combination therapeutic strategy by targeting PRMT5 in R/R MCL patients featured with frequent mutations in *ATM*, *TP53*, and *CDKN2A*. Considering the synergistic effect of the inhibition of both PRMT5 and DDR or cell cycle effector molecules, we anticipate our preclinical study has the translational potential to lead to new clinical trials for MCL.

## Supplementary information

Supplementary Figures and Tables

Table S3

## Data Availability

The datasets used and/or analyzed during the current study are available upon reasonable request from the corresponding author.
