# Correction: Exploiting PRMT5 as a target for combination therapy in mantle cell lymphoma characterized by frequent *ATM* and *TP53* mutations

**DOI:** 10.1038/s41408-026-01472-4

**Published:** 2026-04-08

**Authors:** Yuxuan Che, Yang Liu, Yixin Yao, Holly A. Hill, Yijing Li, Qingsong Cai, Fangfang Yan, Preetesh Jain, Wei Wang, Lixin Rui, Michael Wang

**Affiliations:** 1 Department of Lymphoma and Myeloma, The University of Texas MD Anderson Cancer Center, 1515 Holcombe Blvd., Houston, TX 77030 USA; 2 Department of Bioinformatics and Computer Biology, The University of Texas MD Anderson Cancer Center, 1515 Holcombe Blvd., Houston, TX 77030 USA; 3 School of Biomedical Informatics, University of Texas Health Science Center at Houston, 7000 Fannin Street, Houston, TX 77030 USA; 4 Department of Medicine, the University of Wisconsin-Madison, 1111 Highland Avenue, Madison, WI 53726 USA; 5 Department of Stem Cell Transplantation and Cellular Therapy, The University of Texas MD Anderson Cancer Center, Houston, TX 77030 USA

Correction to: *Blood Cancer Journal* 10.1038/s41408-023-00799-6, published online 17 February 2023

In this article fig. 3D has been updated.

**Figure 3D** (current-with error)
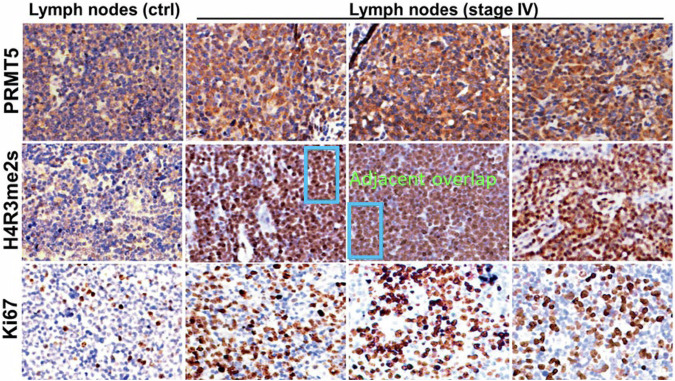


**Figure 3D** (corrected)
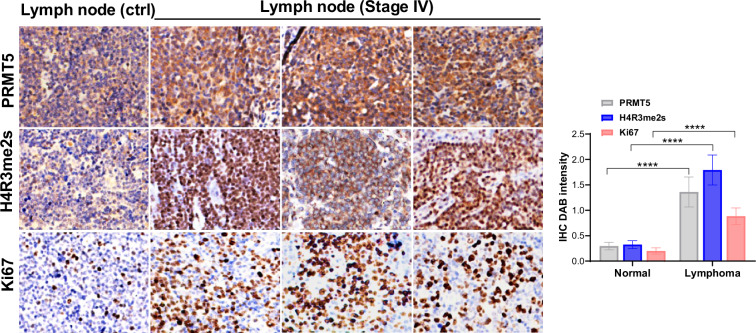


The orginal article has been corrected.

