# Supplementary material for: Exploiting PRMT5 as a target for combination therapy in mantle cell lymphoma characterized by frequent ATM and TP53 mutations
Source: Blood Cancer J. 2023 Feb 17;13(1):27. doi: 10.1038/s41408-023-00799-6 (PMC9935633; doi:10.1038/s41408-023-00799-6)
Supplement: Supplementary file 1 — Supplementary Figures and Tables [file 41408_2023_799_MOESM1_ESM.pdf]

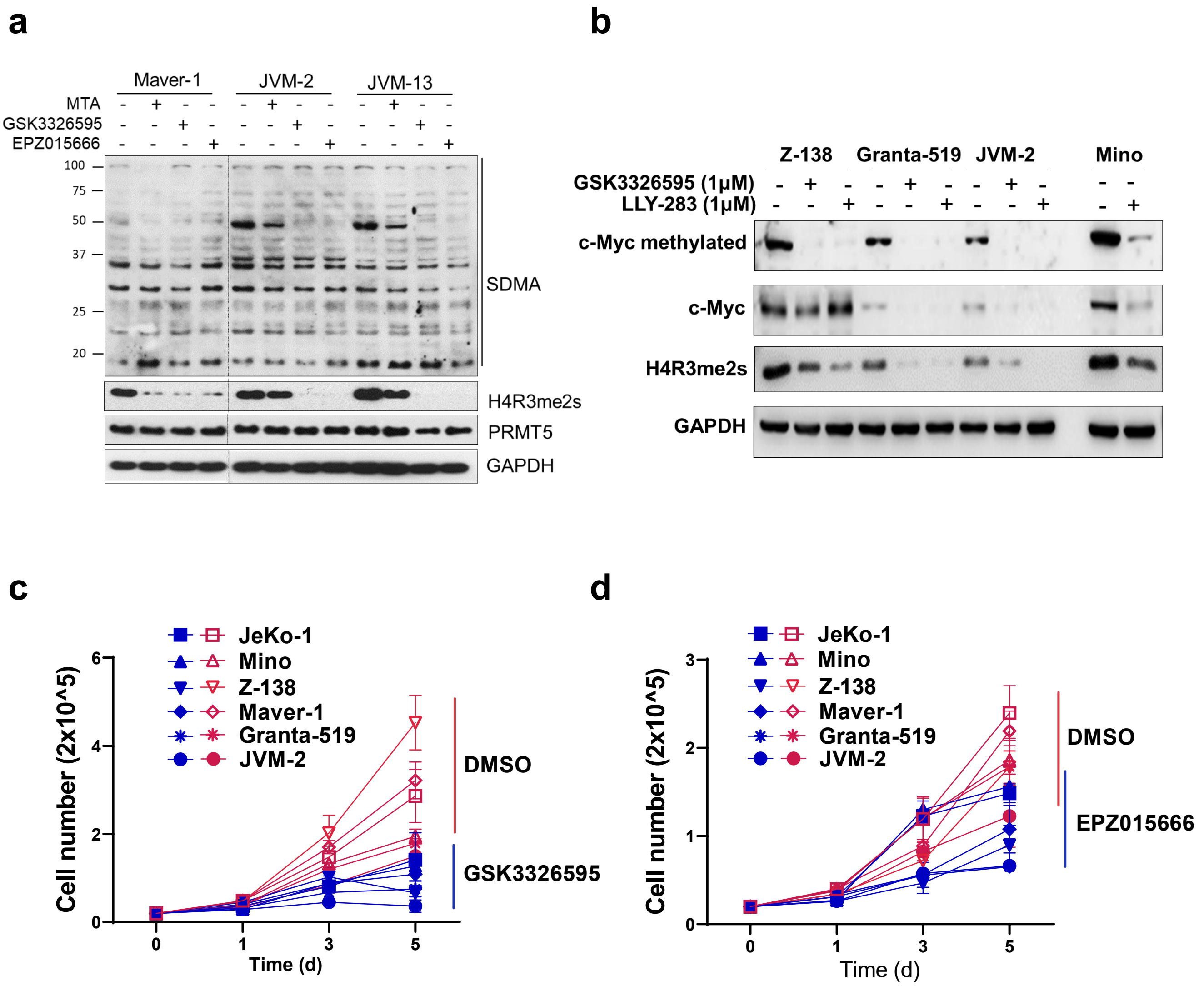

Figure S1. PRMT5 coordinates with multiple oncogenic drivers to promote cell growth and its inhibition suppresses the proliferation of MCL cells.
a. Western-blotting analysis of PRMT5 expression and protein SDMA modification in MCL cell lines (Maver-1, JVM-2, and JVM-13) treated with PRMT5 inhibitors GSK3326595, or EPZ015666. **b**. Western-blotting analysis of the expression of c-Myc and SDMA-modified c-Myc using SDMA-specific antibody, in MCL cell lines (Z-138, Granta-519, JVM-2 and Mino) treated with GSK3326595 or LLY-283. **c**. Proliferation assay of MCL cell lines treated with GSK3326595. **d**. Proliferation assay with MCL cell lines treated with EPZ015666. For all panels: $*p < 0.05$; $**p < 0.01$; $***p < 0.001$.

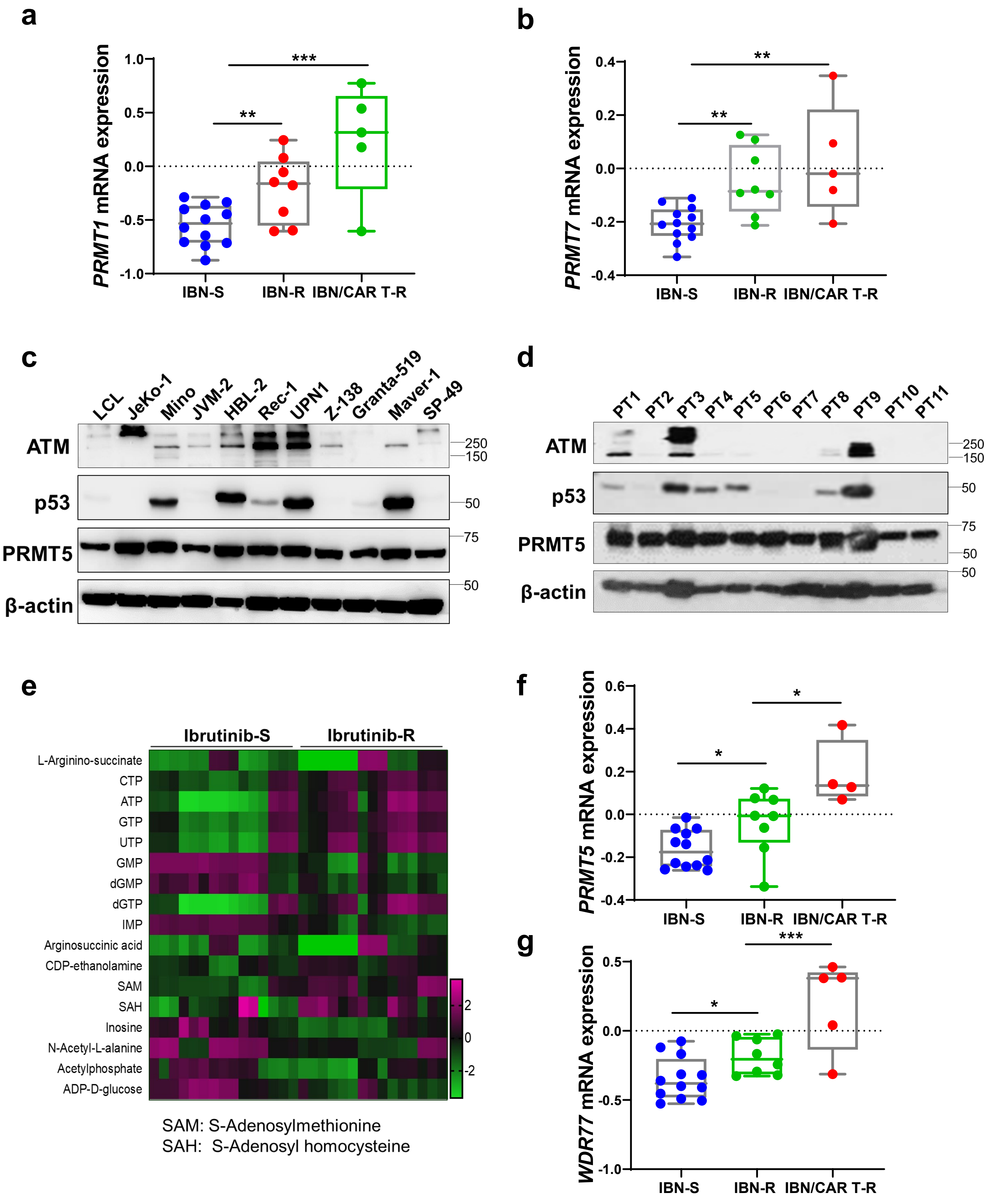
a
PRMT1 mRNA expression
1.0
0.5
0.0
-0.5
-1.0
***
**
IBN-S
IBN-R
IBN/CAR T-R
b
PRMT7 mRNA expression
0.4
0.2
0.0
-0.2
-0.4
**
**
IBN-S
IBN-R
IBN/CAR T-R
c
LCL
JeKo-1
Mino
JVM-2
HBL-2
Rec-1
UPN1
Z-138
Granta-519
Maver-1
SP-49
ATM
p53
PRMT5
β-actin
250
150
50
75
50
d
PT1
PT2
PT3
PT4
PT5
PT6
PT7
PT8
PT9
PT10
PT11
ATM
p53
PRMT5
β-actin
250
150
50
75
50
50
e
Ibrutinib-S
Ibrutinib-R
L-Arginino-succinate
CTP
ATP
GTP
UTP
GMP
dGMP
dGTP
IMP
Arginosuccinic acid
CDP-ethanolamine
SAM
SAH
Inosine
N-Acetyl-L-alanine
Acetylphosphate
ADP-D-glucose
2
0
-2
SAM: S-Adenosylmethionine
SAH: S-Adenosyl homocysteine
f
PRMT5 mRNA expression
0.6
0.4
0.2
0.0
-0.2
-0.4
*
*
IBN-S
IBN-R
IBN/CAR T-R
g
WDR77 mRNA expression
0.5
0.0
-0.5
-1.0
***
*
IBN-S
IBN-R
IBN/CAR T-R

Figure S2. Protein arginine methyltransferase family members are upregulated in relapsed/refractory MCL patients. **a**. PRMT1 or **b**. PRMT7 expression analyzed by single-cell RNA-seq in clinical specimens from patients with MCL resistant to ibrutinib or CD19 CAR T cells. S, sensitive; R, resistant; IBN, ibrutinib. **c**. Protein expression profiling of PRMT5, ATM and p53 in primary specimens from MCL patients. **d**. Protein expression profiling of PRMT5, ATM and p53 in MCL cell lines using western blotting analysis. **e**. Targeted metabolic analysis of PRMT5 activity in ibrutinib-sensitive and -resistant MCL cell lines. PRMT5 **f**. and WDR77 **g**. expression was analyzed by single-cell RNA-seq in clinical specimens from patients with MCL resistant to ibrutinib or CD19 CAR T cells. For all panels: *$p < 0.05$; **$p < 0.01$; ***$p < 0.001$; ****$p < 0.0001$; ns (not statistically significant), $p \geq 0.05$.

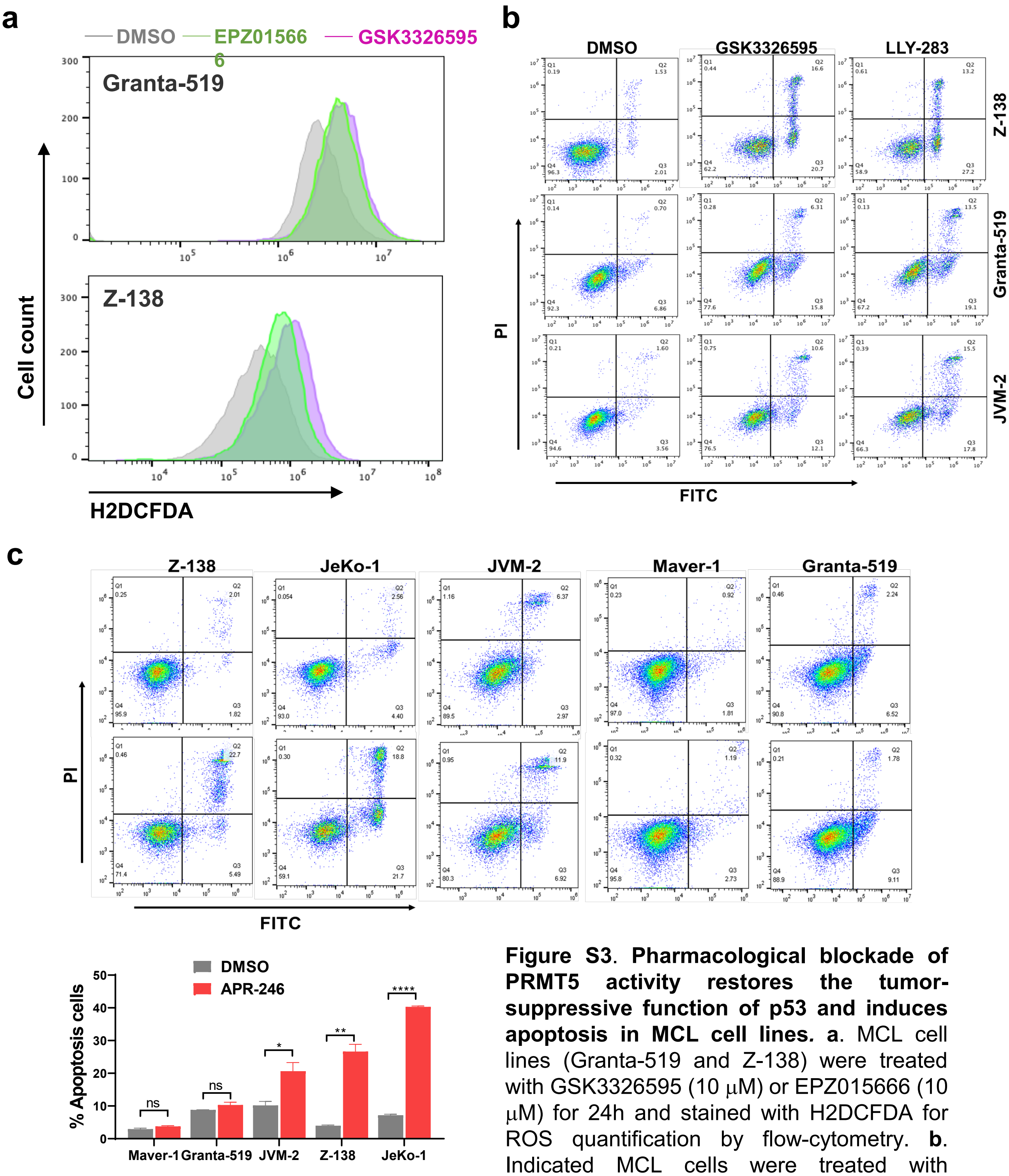

Figure S3. Pharmacological blockade of PRMT5 activity restores the tumor-suppressive function of p53 and induces apoptosis in MCL cell lines. a. MCL cell lines (Granta-519 and Z-138) were treated with GSK3326595 (10 μM) or EPZ015666 (10 μM) for 24h and stained with H2DCFDA for ROS quantification by flow-cytometry. **b**. Indicated MCL cells were treated with GSK3326595 (1 μM) and LLY-283 (1 μM) for 5 days before subjected to apoptosis analysis. **c**. Indicated MCL cell lines were treated with APR-246 (30 μM) for 1-3 d before apoptosis was analyzed by flow cytometry after staining the cells with Annexin V / PI. Bottom: quantitative analysis of apoptosis using Prism. For all panels: *p < 0.05; **p < 0.01; ***p < 0.001; ****p < 0.0001; ns (not statistically significant), p ≥ 0.05.

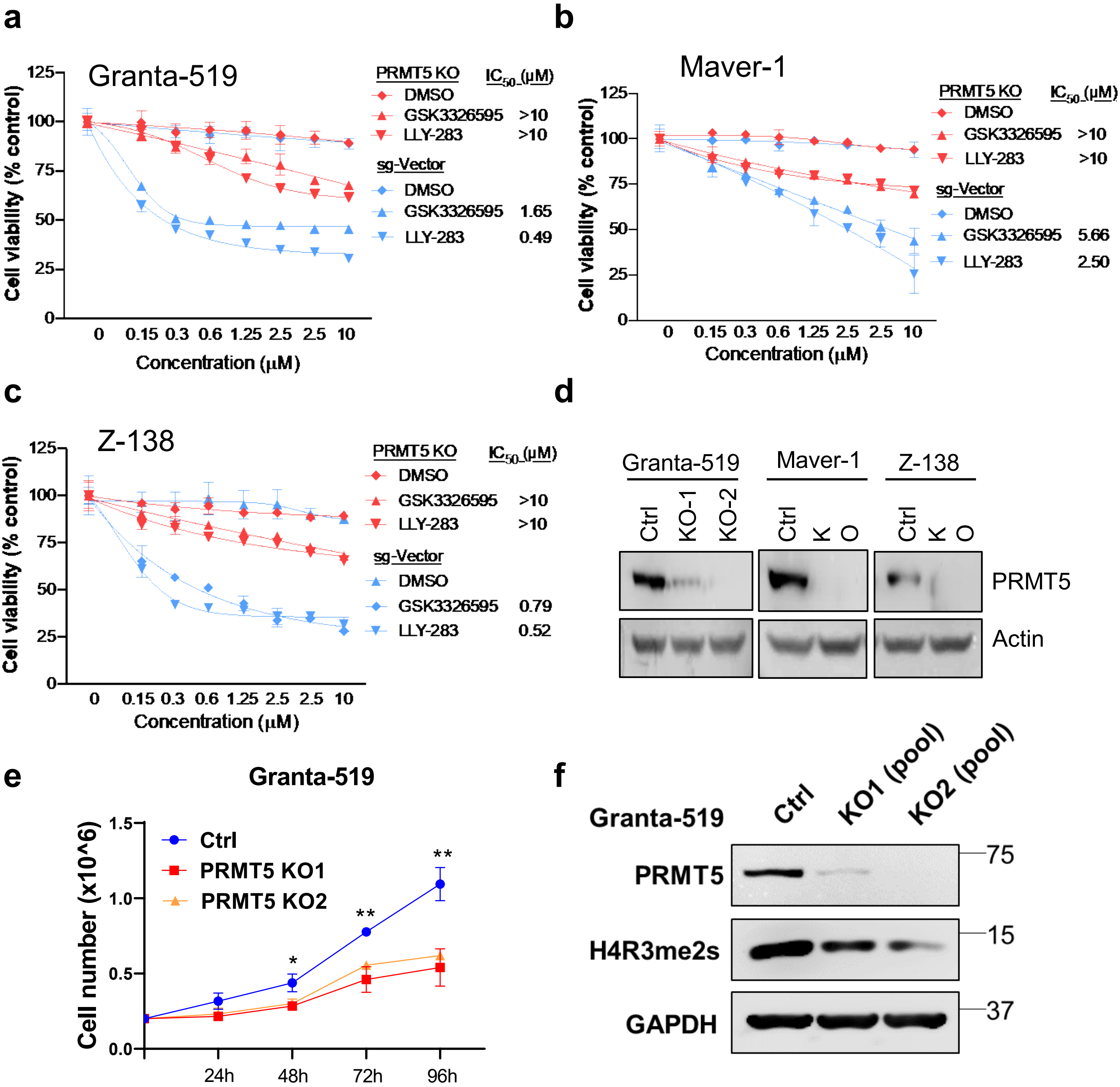

Figure S4. **Genetic ablation of PRMT5 validates the target specificity of the PRMT5 inhibitors. a-c.** Cytotoxicity was assessed in control and PRMT5-knockout MCL cell lines (Granta-519, Maver-1 and Z-138) treated with a 2-fold serial dilution of PRMT5 inhibitors GSK3326595 or LLY-283 for 5 days. **d**. Western-blotting analysis of PRMT5 in the cells used in **a-c**. **e**. Cell proliferation was analyzed in control and PRMT5-knockout Granta-519. **f**. Western-blotting analysis of PRMT5 in the knockout cells used in **e**.

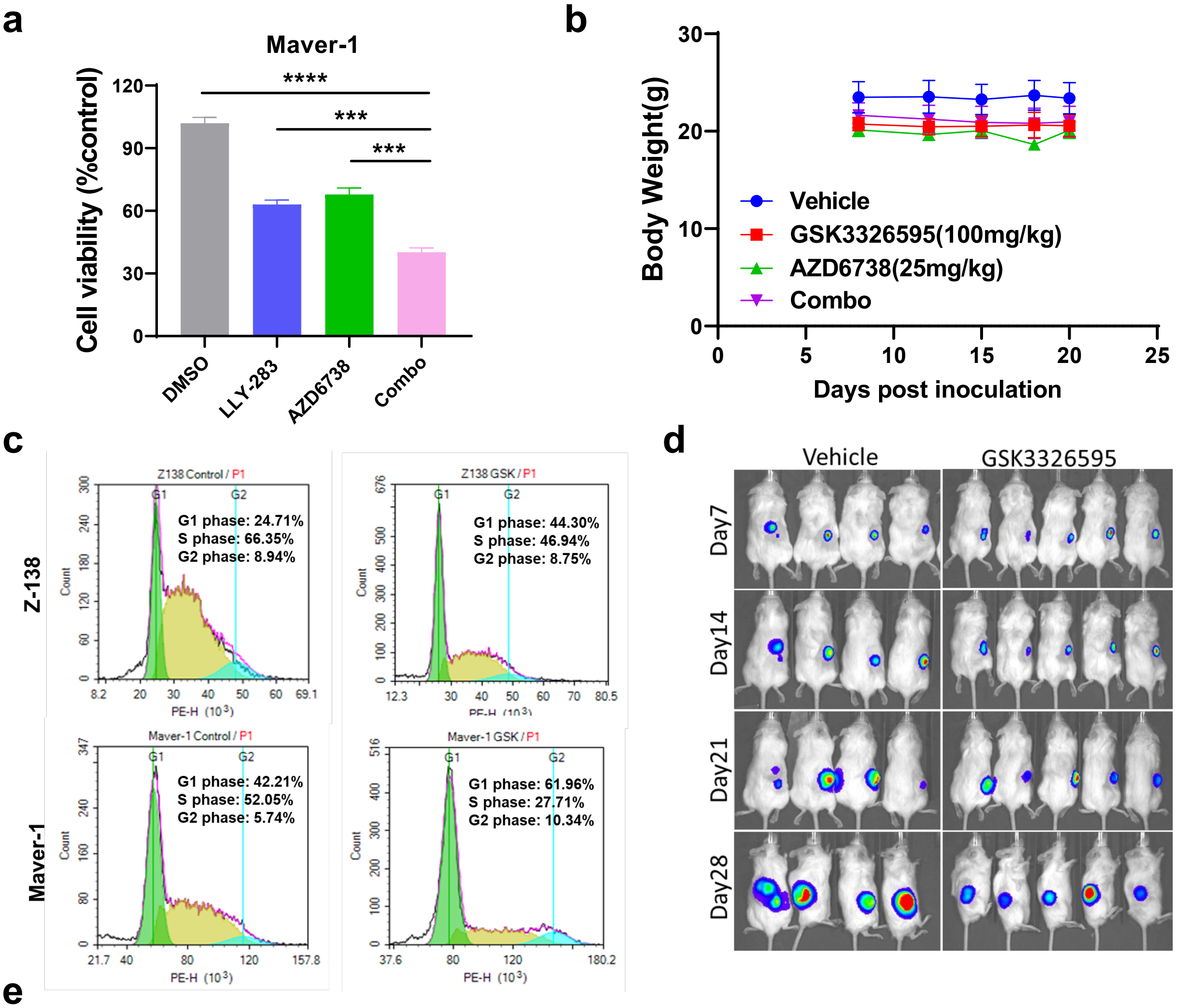

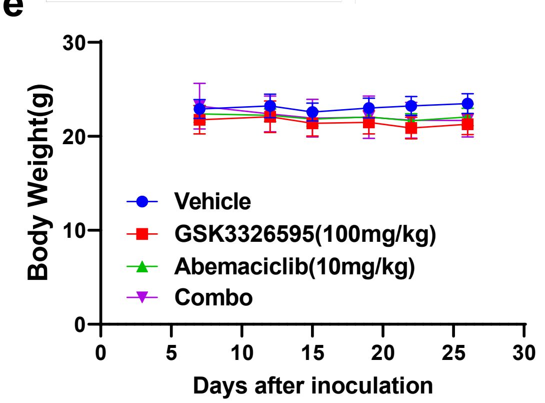

Figure S5. Concurrent targeting of PRMT5, DDR effectors or CDK4 exerts augmented anti-tumor efficacy towards MCL with *ATM* and/or *TP53 or CDKN2A* alterations. a. Cell viability of Maver-1 cells treated with LLY-283, AZD6738, or their combination. *p* value was determined by two-tailed independent Student's *t*-test. **b.** Body weights of NSG mice implanted with Granta-519 and treated with vehicle (n=5), GSK3326595 (100 mg/kg, daily; n=5), AZD6738 (25 mg/kg, daily; n=5), or the combination of both inhibitors n=5 were measured on specified days. **c.** Cell cycle phases of Maver-1 and Z-138 treated with GSK3326595 were analyzed using the Novocyte Flow Cytometer (ACEA Biosciences). **d.** Tumor growth in NSG mouse xenograft implanted with Maver-1 expressing firefly luciferase treated with vehicle or GSK3326595 was monitored on specified days by bioluminescent imaging (n = 5). **e**. Body weights of mice engrafted with Maver-1 treated with vehicle, GSK3326595 (100 mg/kg, daily), abemaciclib (10 mg/kg, daily), or the combination of both inhibitors were measured on specified days. For all panels: *$p < 0.05$; **$p < 0.01$; ***$p < 0.001$; ****$p < 0.0001$; ns: not statistically significant, $p >= 0.05$.

Table S1 Genetic status of *ATM, TP53, CDKN2A* in MCL cell lines

**Cell lines**	***ATM* (Allele 1/Allele 2)**	***TP53* (Allele 1/Allele 2)**	***CDKN2A* (Allele 1/Allele 2)**
Granta-519	Del/Mut (1-4)	WT/Del (4, 5)	Del/Del (6)
JVM-2	WT (3, 4)	WT (3, 4, 7)	WT/WT (8)
Maver-1	Del (9)	Del/Mut (7, 9)	Del (9, 10)
UPN1	WT/Mut (11)	Mut (11)	NR
Z-138	NR, Protein detectable (12)	WT (7, 8)	Heterozygous del (8)
HBL-2	WT/WT (4)	Del/Mut (4)	Heterozygous del (8)
JeKo-1	Proficient (13, 14)	Del (7)	WT/Del (15)
Rec-1	WT (3)	WT/Mut (3, 16)	Del/Del (15)
Mino	Proficient (13, 14)	Mut (7, 14, 17)	Protein detectable (14, 17)
SP-49	Del (18)	NR	NR

Del, deletion; **Mut**, mutation; **WT**, wild type; **NR**: not reported

References

1. Rudolph C, Steinemann D, Von Neuhoff N, Gadzicki D, Ripperger T, Drexler HG, et al. Molecular cytogenetic characterization of the mantle cell lymphoma cell line GRANTA-519. Cancer Genet Cytogenet. 2004;153(2):144-50.
2. Vorechovský I, Luo L, Dyer MJ, Catovsky D, Amlot PL, Yaxley JC, et al. Clustering of missense mutations in the ataxia-telangiectasia gene in a sporadic T-cell leukaemia. Nat Genet. 1997;17(1):96-9.
3. Ferrer A, Marcé S, Bellosillo B, Villamor N, Bosch F, López-Guillermo A, et al. Activation of mitochondrial apoptotic pathway in mantle cell lymphoma: high sensitivity to mitoxantrone in cases with functional DNA-damage response genes. Oncogene. 2004;23(55):8941-9.
4. Williamson CT, Kubota E, Hamill JD, Klimowicz A, Ye R, Muzik H, et al. Enhanced cytotoxicity of PARP inhibition in mantle cell lymphoma harbouring mutations in both ATM and p53. EMBO Mol Med. 2012;4(6):515-27.
5. Camps J, Salaverria I, Garcia MJ, Prat E, Beà S, Pole JC, et al. Genomic imbalances and patterns of karyotypic variability in mantle-cell lymphoma cell lines. Leuk Res. 2006;30(8):923-34.
6. Jadayel DM, Lukas J, Nacheva E, Bartkova J, Stranks G, De Schouwer PJ, et al. Potential role for concurrent abnormalities of the cyclin D1, p16CDKN2 and p15CDKN2B genes in certain B cell non-Hodgkin's lymphomas. Functional studies in a cell line (Granta 519). Leukemia. 1997;11(1):64-72.
7. Yoshimura M, Ishizawa J, Ruvolo V, Dilip A, Quintás-Cardama A, McDonnell TJ, et al. Induction of p53-mediated transcription and apoptosis by exportin-1 (XPO1) inhibition in mantle cell lymphoma. Cancer Sci. 2014;105(7):795-801.
8. Tucker CA, Bebb G, Klasa RJ, Chhanabhai M, Lestou V, Horsman DE, et al. Four human t(11;14)(q13;q32)-containing cell lines having classic and variant features of Mantle Cell Lymphoma. Leuk Res. 2006;30(4):449-57.
9. Zamò A, Ott G, Katzenberger T, Adam P, Parolini C, Scarpa A, et al. Establishment of the MAVER-1 cell line, a model for leukemic and aggressive mantle cell lymphoma. Haematologica. 2006;91(1):40-7.
10. Beà S, Salaverria I, Armengol L, Pinyol M, Fernández V, Hartmann EM, et al. Uniparental disomies, homozygous deletions, amplifications, and target genes in mantle cell lymphoma revealed by integrative high-resolution whole-genome profiling. Blood. 2009;113(13):3059-69.
11. M'Kacher R, Bennaceur A, Farace F, Laugé A, Plassa LF, Wittmer E, et al. Multiple molecular mechanisms contribute to radiation sensitivity in mantle cell lymphoma. Oncogene. 2003;22(39):7905-12.
12. Williamson CT, Muzik H, Turhan AG, Zamò A, O'Connor MJ, Bebb DG, et al. ATM deficiency sensitizes mantle cell lymphoma cells to poly(ADP-ribose) polymerase-1 inhibitors. Mol Cancer Ther. 2010;9(2):347-57.
13. Sarkar A, Stellrecht CM, Vangapandu HV, Ayres M, Kaipparettu BA, Park JH, et al. Ataxia-telangiectasia mutated interacts with Parkin and induces mitophagy independent of kinase activity. Evidence from mantle cell lymphoma. Haematologica. 2021;106(2):495-512.
14. Amin HM, McDonnell TJ, Medeiros LJ, Rassidakis GZ, Leventaki V, O'Connor SL, et al. Characterization of 4 mantle cell lymphoma cell lines. Arch Pathol Lab Med. 2003;127(4):424-31.
15. Marcé S, Balagué O, Colomo L, Martinez A, Höller S, Villamor N, et al. Lack of methylthioadenosine phosphorylase expression in mantle cell lymphoma is associated with shorter survival: implications for a potential targeted therapy. Clin Cancer Res. 2006;12(12):3754-61.
16. Erazo T, Evans CM, Zakheim D, Chu KL, Refermat AY, Asgari Z, et al. TP53 mutations and RNA-binding protein MUSASHI-2 drive resistance to PRMT5-targeted therapy in B-cell lymphoma. Nat Commun. 2022;13(1):5676.
17. Lai R, McDonnell TJ, O'Connor SL, Medeiros LJ, Oudat R, Keating M, et al. Establishment and characterization of a new mantle cell lymphoma cell line, Mino. Leuk Res. 2002;26(9):849-55.
18. Kawamata N, Ogawa S, Gueller S, Ross SH, Huynh T, Chen J, et al. Identified hidden genomic changes in mantle cell lymphoma using high-resolution single nucleotide polymorphism genomic array. Exp Hematol. 2009;37(8):937-46.

Table S2. Sequences of primers and sgRNA used in this study

Gene name	Primer sequence
MDM2	Forward: 5'-CCCAAGACAAAGAAGAGAGTGTGG-3'<br>Reverse: 5'-CTGGGCAGGGCTTATTCCTTTTCT-3'
P21	Forward: 5'-CTGAGACTCTCAGGGTCGAA-3'<br>Reverse: 5'-CGGCGTTTGGAGTGGTAGAA-3'
PUMA	Forward: 5'-ACGACCTCAACGCACAGTACGA-3'<br>Reverse: 5'-GTAAGGGCAGGAGTCCCATGATGA-3'
AR	Forward: 5'-GTGGAAGCTGCAAGGTCTTC-3'<br>Reverse: 5'-CGAAGACGACAAGATGGACA-3'
DNAPK	Forward: 5'-GAACCTTTCATCAAACGAAGCAATATCC-3'<br>Reverse: 5'-CTGAGGACGTGACTGTCAGAAG-3'
NHEJ1	Forward: 5'-CAAGGCGCTGGAGATCCTC-3'<br>Reverse: 5'-CTGAAGTACCCGTGGACTCTTTC-3'
RAD51	Forward: 5'-GTTGGGACTACAGGTGGAATTGAG-3'<br>Reverse: 5'-CAATGGGAAGCTGGCAGGTG-3'
MDM4	Forward: 5'-TGTGGT GGAGATCTTTTGGG-3'<br>Reverse: GCAGTGTGGGGATATCGT-3'
PRMT5	**sgRNA sequence**
Knockout_1	Forward: 5'-ATACAGCTTTATCCGCCGGT-3'<br>Reverse: 5'-ACCGGCGGATAAAGCTGTAT-3'
Knockout_2	Forward: 5'-AGTTCATAGGCATTAGGTGG-3'<br>Reverse: 5'-CCACCTAATGCCTATGAACT-3'
